# Complete regression of hip structural damage with anakinra: a case report

**DOI:** 10.1186/1546-0096-12-S1-P347

**Published:** 2014-09-17

**Authors:** Francisca Aguiar, Joana Abelha-Aleixo, Mariana Rodrigues, Iva Brito

**Affiliations:** 1 Pediatric Rheumatology Unit, São João Hospital, Oporto, Portugal; 2 Rheumatology, São João Hospital, Oporto, Portugal

## Introduction

Systemic juvenile idiopathic arthritis (SJIA) is an autoinflammatory rheumatic disease characterized by the presence of intermittent spiking fever, evanescent erythematous rash, arthritis, lymphadenopathy, hepatosplenomegaly, serositis and elevated inflammatory markers. It accounts for only 10-20% of all patients with JIA but is associated with higher morbidity and mortality. Despite the variety of treatments available, in some cases the disease is difficult to control and has a refractory course. The recent knowledge of the key role of certain cytokines (interleukin (IL)-1, IL-6 and IL-18) in its pathogenesis makes them potential therapeutic targets. Anakinra is a recombinant form of human IL-1 receptor antagonist (IL-1Ra) that has been used in SJIA refractory cases and as first line therapeutic agent in selected patients.

## Objectives

To describe a case of clinical and imagiological complete remission with Anakinra treatment in a patient with SJIA and severe coxitis.

## Methods

The authors report the case of a fourteen-year-old Caucasian girl diagnosed with SJIA by the age of four, when she presented with prolonged febrile syndrome, polyarthritis, evanescent salmon pink rash and pericardial effusion. She was initially treated with oral corticosteroids (prednisolone 1 mg/kg/day). In spite of clinical improvement, systemic and articular activity of the disease persisted with 1-2 exacerbations a year and in the beginning of the year 2007 she started subcutaneous methotrexate (10 mg/week). Despite methotrexate treatment escalation, the patient developed severe left coxitis with major limitation of hip joint mobility, which did not respond to triamcinolone hexacetonide injection.

## Results

Due to the persistence of arthritis and elevated inflammatory markers she started Anakinra (1 mg/kg/day) in July 2009. Since then there has been sustained improvement with resolution of clinical symptoms, normalization of laboratory parameters and complete clinical and radiological resolution of coxitis, which allowed discontinuation of methotrexate and corticosteroids. Anakinra was well tolerated and there were no adverse events. The patient achieved prolonged remission.

## Conclusion

A significant number of patients with SJIA has persistent disease despite the treatments used. In this case we report the therapeutic success with Anakinra in a patient with refractory systemic and articular manifestations emphasizing complete regression of structural damage of the hip joint.

## Disclosure of interest

None declared

